# Development and validation of a deep learning model for liver shear stiffness regression using abdominal multiparametric MRI across multiple sites and vendors

**DOI:** 10.1007/s00330-026-12448-0

**Published:** 2026-05-13

**Authors:** Redha Ali, Hailong Li, Scott B. Reeder, David Harris, William Masch, Anum Aslam, Krishna P. Shanbhogue, Nehal A. Parikh, Lili He, Jonathan R. Dillman

**Affiliations:** 1 Imaging Research Center, Department of Radiology, Cincinnati Children’s Hospital Medical Center, Cincinnati, OH USA; 2 Department of Radiology, University of Cincinnati College of Medicine, Cincinnati, OH USA; 3 Neurodevelopmental Disorders Prevention Center, Perinatal Institute, Cincinnati Children’s Hospital Medical Center, Cincinnati, OH USA; 4 Artificial Intelligence Imaging Research Center, Cincinnati Children’s Hospital Medical Center, Cincinnati, OH USA; 5 Department of Radiology, University of Wisconsin, Madison, WI USA; 6 Departments of Medical Physics, Biomedical Engineering, Medicine, and Emergency Medicine, University of Wisconsin, Madison, WI USA; 7 Department of Radiology, Michigan Medicine, Ann Arbor, MI USA; 8 Department of Radiology, New York University, New York, NY USA; 9 Department of Pediatrics, University of Cincinnati College of Medicine, Cincinnati, OH USA; 10 Department of Computer Science, Biomedical Engineering, Biomedical Informatics, Cincinnati Children’s Hospital Medical Center, Cincinnati, OH USA

**Keywords:** Liver stiffness, Deep learning, Magnetic resonance imaging, Magnetic resonance elastography, Liver diseases, Chronic

## Abstract

**Objective:**

Liver stiffness measurement is important for assessing chronic liver disease (CLD). MR elastography (MRE) requires specialized hardware and expertise. Non-invasive deep learning (DL) models using multiparametric abdominal MRI may provide an accessible alternative. We sought to develop and validate a DL model for predicting continuous liver shear stiffness from non-contrast multiparametric abdominal MRI and electronic health record (EHR) data across multiple sites and vendors.

**Materials and methods:**

This was a retrospective, multi-institutional study. We analyzed 3680 abdominal MRI examinations from 3376 patients with confirmed or suspected CLD. Non-contrast T1-weighted (T1w), T2-weighted (T2w), and diffusion-weighted imaging (DWI) with EHR data were used as inputs. MRE-derived liver shear stiffness served as the reference. A transformer-based multi-channel DL model was trained using multi-site 10-fold cross-validation and evaluated on temporally held-out internal (*n* = 1224) and independent external (*n* = 365) test sets. Performance was measured by Pearson’s correlation coefficient (r); residual analysis assessed bias.

**Results:**

In cross-validation, the model achieved an r of 0.78 (95% CI: 0.75, 0.80). On the internal test set, r was 0.77 (95% CI: 0.73, 0.80), and on the external set, r was 0.76 (95% CI: 0.69, 0.83). The model showed no significant bias based on age, sex, or BMI (*p* > 0.05). In patients with and without steatotic liver disease, r was 0.74 and 0.76, respectively.

**Conclusion:**

Our transformer-based multi-channel model predicts continuous liver shear stiffness from routinely acquired multiparametric MRI and EHR data with moderate correlation to MRE, representing a potential step toward accessible, non-invasive liver stiffness estimation.

**Key Points:**

***Question*** *Can routinely acquired multiparametric abdominal MRI and electronic health record data predict liver stiffness across multiple sites and scanner vendors using a deep learning approach?*

***Findings**** The optimized deep learning model predicted liver stiffness with r = 0.78 in cross-validation and r = 0.76 in external validation using multiparametric MRI and electronic health record data*.

***Clinical relevance**** This study introduces a preliminary yet robust AI method to estimate liver stiffness from routine multiparametric MRI and EHR data, offering a scalable fibrosis assessment approach suitable for opportunistic evaluation and as a complementary tool when MRE is unavailable*.

**Graphical Abstract:**

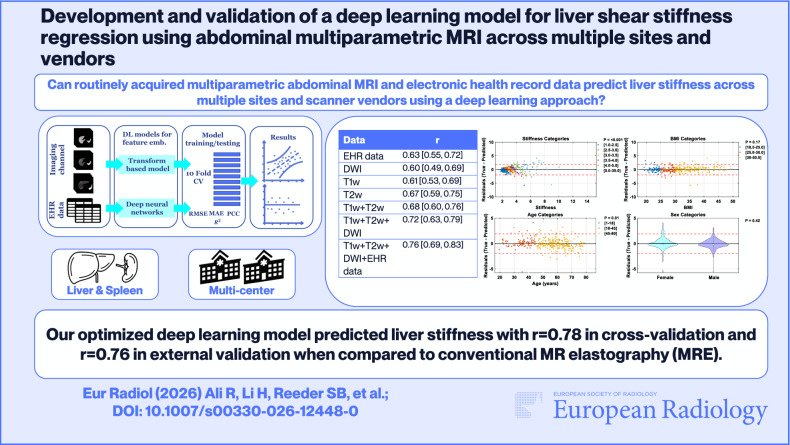

## Introduction

Chronic liver disease (CLD) represents a significant global health issue, contributing to both morbidity and mortality worldwide [1, 2]. If left untreated, liver fibrosis from CLD can progress to cirrhosis, increasing the risk of hepatocellular carcinoma, liver failure, and other life-threatening complications [3, 4]. Assessing the severity of liver fibrosis is crucial, as it guides treatment and monitoring, and is the best-known predictor of patient outcomes in patients with CLD [5].

Liver biopsy with histological analysis is the current gold standard for detecting and staging liver fibrosis [6]. However, this invasive diagnostic method has several limitations, including sampling errors, low acceptance by patients (and their parents or guardians in the case of children), substantial inter-pathologist variability, high cost, and the risk of intra-abdominal bleeding [7, 8]. Therefore, there is an urgent need for reliable, non-invasive methods to accurately assess fibrosis severity, improving comfort, reducing risks, and enabling a more reliable and cost-effective diagnosis [9].

The aspartate aminotransferase-to-platelet ratio index (APRI) and fibrosis-4 (FIB-4) score, which are based on laboratory blood testing, are commonly used to evaluate liver fibrosis. Unfortunately, these metrics are limited by suboptimal diagnostic performance and insufficient sensitivity, particularly for intermediate stages of fibrosis [10, 11]. Magnetic resonance elastography (MRE) and ultrasound (US) shear-wave elastography (SWE) are increasingly recognized as promising non-invasive tools for assessing patients with CLD [12–14]. Both MRI and US can reliably distinguish between absent or early-stage fibrosis and advanced liver fibrosis in pediatric and adult populations [14–19]. However, US SWE results can vary significantly based on the operator’s experience, technique, and the US system and transducer employed [20, 21]. This variability can lead to inconsistent assessments, especially in less experienced hands. On the other hand, MRE requires costly specialized equipment and software as well as skilled operators. These resources may not be accessible in resource-limited settings [22]. Furthermore, MRE can be adversely impacted by confounding factors like large body habits, iron overload, and motion artifacts, which can compromise the usability of its results [23]. Therefore, there remains a pressing need to develop more accessible, cost-effective, and accurate non-invasive methods for assessing liver fibrosis that can be widely implemented across diverse healthcare settings, regardless of operator experience or regional resource limitations.

Recently, routine clinical MRI images have been applied to predict or categorize the severity of liver stiffening using machine learning (ML) and deep learning (DL) algorithms [15, 24–26]. Pollack et al [25] developed a DL model to predict voxel-wise liver stiffness using traditional T1-weighted (T1w) and T2-weighted (T2w) MR images. Ali et al [26] developed a DL-based model to stratify the severity of liver stiffness in a diverse, large, multicenter combined pediatric and adult cohort using combined T1w and T2w MR image. Using T1w and T2w MR images, these prior studies have demonstrated areas under the receiver operating characteristic curve (AUROCs) between 0.64 and 0.86 for categorizing the severity of liver stiffening across multiple studies [15, 24, 25] and a Pearson’s correlation coefficient (r) of 0.50 for predicting continuous liver shear stiffness [25]. These results suggest that T1w and T2w imaging have the potential to assess liver stiffness, but their current predictive accuracy varies widely across studies, and these sequences alone may not be sufficient for precise liver stiffness quantification. Meanwhile, studies have shown that diffusion-weighted imaging (DWI) data might also be promising in liver fibrosis assessment, since a strong correlation was observed between tissue water diffusivity and liver tissue elasticity (${R}^{2}\,$= 0.81) [27, 28].

Given the predictive potential of DWI and the existing evidence supporting the use of T1w and T2w MR images for liver stiffness prediction, the purpose of this work is to combine T1w, T2w, and DWI data to develop an enhanced model for continuous liver shear stiffness estimation. Specifically, we developed a transformer-based multi-channel DL model that uses routinely available, non-contrast multiparametric MRI sequences (T1w, T2w, and DWI) as well as electronic health record (EHR) data from both pediatric and adult CLD patients across multiple institutions and MRI vendors. Additionally, we considered the potential impact of confounding factors, such as patient age, biologic sex, and presence of hepatic steatosis, on predictive accuracy across varying patient profiles. Finally, we conducted a comprehensive set of experiments to validate our final optimized DL model.

## Materials and methods

This retrospective, multicenter study received institutional review board approval at all four participating institutions (Cincinnati Children’s Hospital Medical Center [CCHMC], New York University [NYU], the University of Wisconsin [UW], and the University of Michigan [UM]). Informed consent (and informed assent, as appropriate) was waived due to the retrospective nature of the study, and all imaging and EHR data were anonymized and securely stored before being shared between sites, complying with the Health Insurance Portability and Accountability Act (HIPAA).

### Study participants and MR image acquisition

**Fig. 1:**
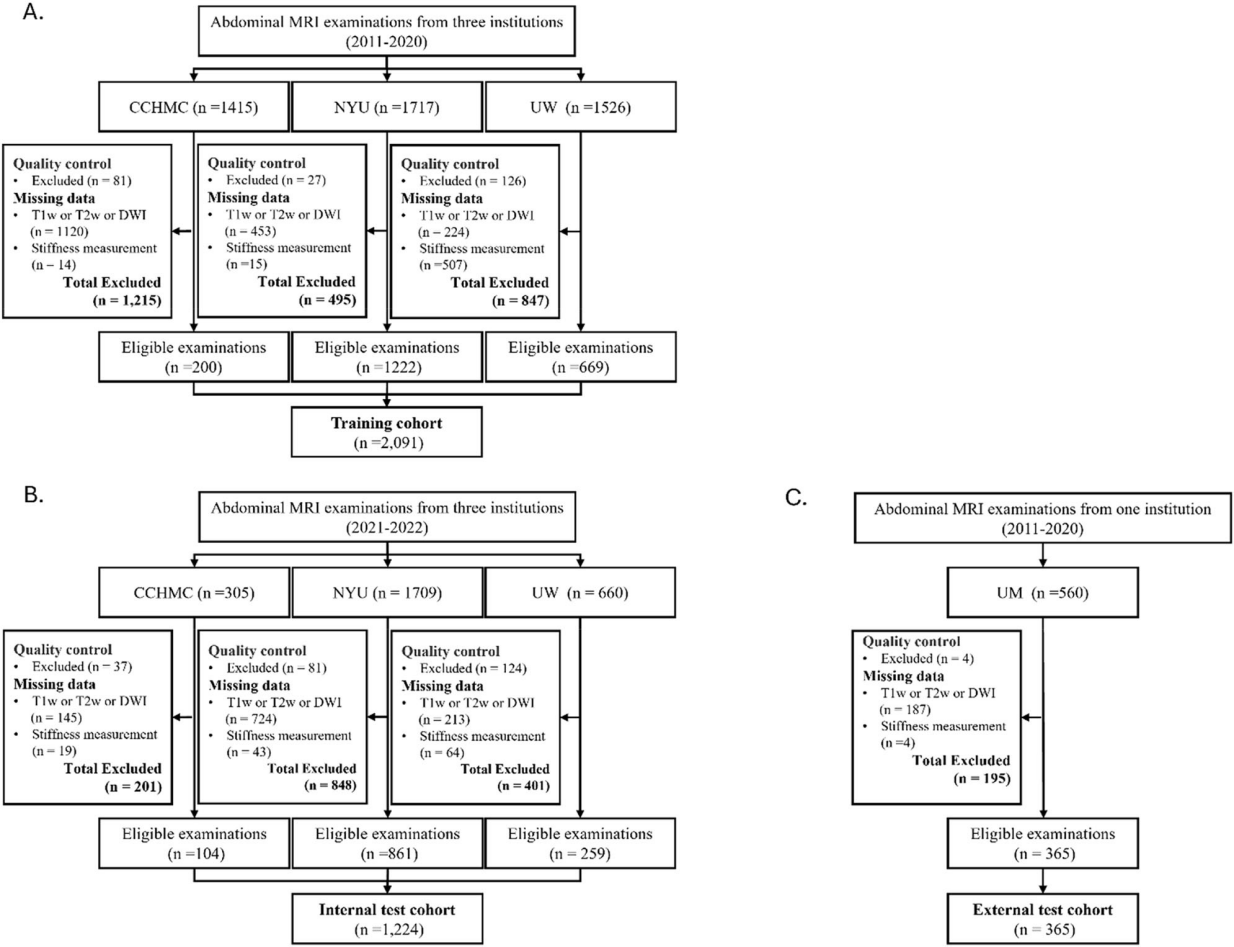
Study cohort selection flowchart. **A** Selection process for the training cohort from abdominal MRI examinations collected between 2011 and 2020 at three institutions (Cincinnati Children’s Hospital Medical Center (CCHMC), New York University (NYU), and the University of Wisconsin (UW)), highlighting quality control exclusions and missing data, with a final total of 2091 eligible examinations. **B** Selection process for the internal test cohort from abdominal MRI examinations collected between 2021 and 2022 at the same three institutions, with 1224 eligible examinations after exclusions. **C** Selection process for the external test cohort using data from a separate institution (University of Michigan (UM)) collected between 2011 and 2020, resulting in 365 eligible examinations after exclusions

We searched the Picture Archiving and Communication Systems (PACS) of all institutions to identify patients who underwent clinical abdominal MRI exams that included MRE performed between 2011 and 2022 (Fig. 1). All patients were required to have undergone liver shear stiffness measurement using MRE at the same time as the clinical MRI acquisition of non-contrast three-dimensional (3D) T1w gradient echo fat-suppressed (e.g., LAVA, VIBE, THRIVE, or water-only Dixon), axial T2w fast spin-echo fat-suppressed, and axial DWI spin-echo echo-planar sequences. The following patients were excluded: (1) MRI exams missing T1w, T2w, and/or DWI MR images, (2) lack of clinical liver shear stiffness measurements in associated imaging reports, and/or (3) severe artifacts affecting T1w, T2w, and/or DWI images. Two image analysts conducted a manual artifact review of all study images under the guidance of an experienced radiologist. MRI protocol parameters, MRE acquisition details, and EHR inclusion/exclusion criteria are in Supplementary Materials S.1.1–S.1.3.

### Study overview

**Fig. 2:**
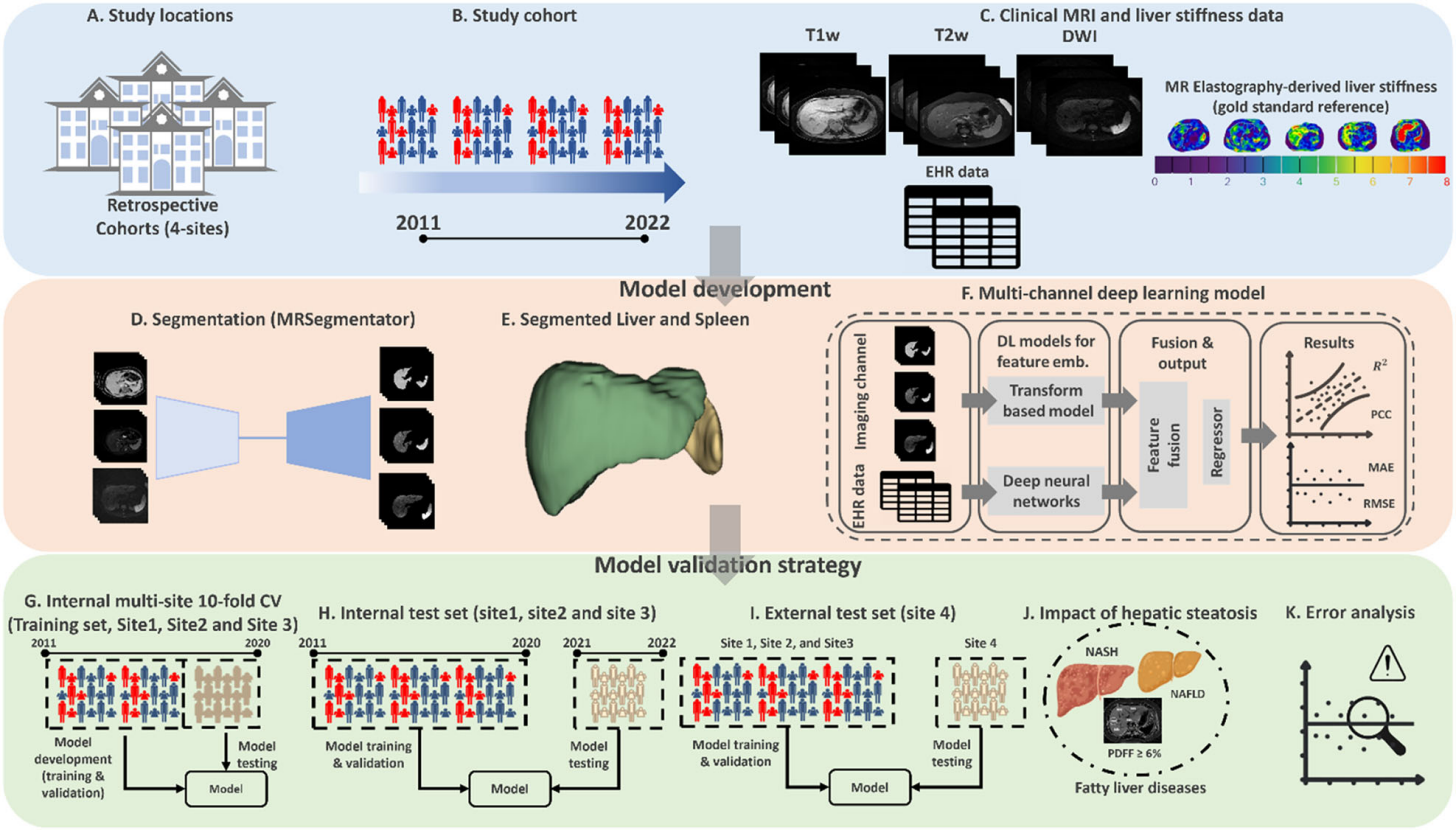
Overview of the study design, model development, and validation strategy. This retrospective multi-institutional study included individuals who underwent abdominal MRI with MR elastography between 2011 and 2022. **A** MRI data were collected from four study sites. **B** The study cohort comprised patients scanned over the study period. **C** Clinical MRI inputs included axial T1w, T2w, and DWI sequences, together with EHR data; MR elastography-derived liver stiffness served as the reference standard. **D** Liver and spleen segmentation were performed using MR Segmentator. **E** Segmented liver and spleen masks were generated from the MRI data for downstream analysis. **F** A multi-channel deep learning framework incorporated imaging channels and EHR data, with feature extraction using transformer-based and deep neural network models, followed by feature fusion and regression to predict liver shear stiffness. Model performance was evaluated using correlation-based and error-based metrics, including the coefficient of determination (*R*^2^), Pearson correlation coefficient (*r*), mean absolute error (MSE), and root mean squared error (MAE). **G** Internal multi-site 10-fold cross-validation was performed using data from Sites 1, 2, and 3 for model development. **H** Internal testing was conducted on a temporally held-out dataset from Sites 1, 2, and 3. **I** External validation was performed using an independent held-out dataset from Site 4. **J** The impact of hepatic steatosis on model performance was assessed by comparing patients with and without fatty liver disease, including nonalcoholic steatohepatitis and nonalcoholic fatty liver disease, defined by proton density fat fraction (PDFF) of 6% or greater. **K** Error analysis was performed to identify model limitations, sources of bias, and factors associated with prediction error

Figure 2 presents an overview of our study. Eligible patients imaged at the four participating institutions between 2011 and 2022 were included in this study (Fig. 2A, B). For each patient, we retrieved routinely acquired clinical non-contrast conventional T1w, T2w, and DWI MRI and associated EHR data (Fig. 2C). The reference standard for our study was clinically reported liver shear stiffness measurements as recorded in the EHR (Fig. 2C). We first used MRSegmentator [29, 30] to segment the liver and spleen from the input sequences (Fig. 2D, E). We then trained a multi-channel supervised regression network with a Swin Transformer-based encoder [31] initialized from pre-trained weights and fine-tuned end-to-end to estimate continuous liver shear stiffness (Fig. 2F). Model performance was evaluated through extensive internal cross-validation and external validation (Fig. 2G–I). We further examined the influence of steatotic liver disease (SLD; previously called nonalcoholic fatty liver disease [NAFLD] and nonalcoholic steatohepatitis [NASH]) on prediction accuracy (Fig. 2J) and assessed potential bias across demographic subgroups and degrees of liver stiffening using Bland–Altman analyses (Fig. 2K).

### Multi-channel deep learning model

Image segmentation and preprocessing are described in Supplementary Methods S.2.1. Model architecture and optimization details are in Supplementary Methods S.2.2.

### Model validation strategy

Participants from four sites were divided into three non-overlapping datasets: a training set of 2091 MRI scans from CCHMC, NYU, and UW (2011–2020), a temporally held-out internal test set of 1224 scans from the same sites (2021–2022), and an independent external test set of 365 scans from UM (2011–2020). The multi-channel model was developed and optimized on the training set using 10-fold cross-validation stratified by site, with each fold comprising 80% training, 10% validation, and 10% testing. The finalized model was then evaluated on the internal test set to simulate prospective performance and on the external test set in a leave-one-site-out design to assess generalizability to new institutional data.

### Hepatic steatosis

We evaluated the performance of DL models in patients without and with SLD. Previous studies have demonstrated that liver fat can impact liver shear stiffness measurements [32, 33]. In this current investigation, we defined the presence of SLD (i.e., MASLD/MASH) as an MRI proton density fat fraction (PDFF) ≥ 6% [34] or a diagnosis of MASLD or MASH recorded in the EHR. Our dataset was divided into two subsets: patients with SLD (*n* = 2118 MRI examinations) and individuals without SLD (*n* = 1562 MRI examinations). Separate training and testing procedures were conducted for each group using a multi-site 10-fold CV.

### Statistical analysis

Model performance was evaluated using Root Mean Squared Error (RMSE), Mean Absolute Error (MAE), Pearson’s correlation coefficient (r), and the coefficient of determination (*R*^2^), each with 95% confidence intervals. A *p*-value < 0.05 was considered statistically significant for inference testing. Analyses were performed using the statistical package of MATLAB 2018a (MathWorks). We performed descriptive analyses, reporting means (standard deviation) for continuous variables and counts and percentages for categorical variables. The two-sided Student’s *t*-test (continuous data) and chi-square test (categorical data) were used to compare baseline differences between cohorts and model performance. Additionally, bias due to patient demographics and phenotypes was evaluated by calculating *p*-values using Analysis of Variance (ANOVA). Four variables, each divided into multiple categories, were considered to assess bias in the predicted liver stiffness values: true average stiffness (1.0–2.5 kPa, 2.5–3.0 kPa, 3.0–3.5 kPa, 3.5–4.0 kPa, 4.0–5.0 kPa, 5.0–30.0 kPa), BMI (18.5–25.0, 25.0–30.0, 30.0–50.0), age (1–18 years, 18–45 years, 45–80 years), and sex (Male, Female). Residuals (the difference of measured liver shear stiffness and predicted stiffness) were calculated for each category within each variable, and *p*-values were determined to evaluate whether differences between categories were statistically significant. A *p*-value greater than 0.05 indicated no detectable bias, while a *p*-value less than 0.05 suggested significant bias.

## Results

### Study sample

This retrospective study involved 3680 MRI scans from 3376 pediatric and adult patients with confirmed or suspected CLD from four institutions. Our study included imaging data obtained from 20 different MRI systems manufactured by GE HealthCare, Philips Healthcare, and Siemens Healthineers, respectively, with field strengths including both 1.5 T and 3 T. The study population had a mean (SD) age of 50.1 (16.5) years and a mean BMI of 29.1 (6.6) kg/m², with 1924 (52.3%) female patients. The participant selection process and inclusion/exclusion criteria are summarized in Fig. 1, and additional study sample characteristics are detailed in Table 1.

**Table 1:** Participant characteristics, including demographic information and liver shear stiffness characteristics for training and external test cohorts

		Training set	Internal test set	External test set
No. of patients		1876	1135	365
No. of examinations		2091	1224	365
	Female, *n* (%)	984 (52.4)	601 (53.0), *p* = 0.79	185 (50.7), *p* = 0.53
	Male, *n* (%)	892 (47.6)	534 (47.0), *p* = 0.79	180 (49.3), *p* = 0.53
Age (years)		41.8 ± 12.5	53.9 ± 13.9, *p* = 0.40	41.6 ± 12.6, *p* < 0.001
Weight (kg)		78.9 ± 21.9	81.5 ± 19.2, *p* < 0.001	78.6 ± 19.0, *p* < 0.001
Height (cm)		166.0 ± 12.2	168.1 ± 8.6, *p* = 0.51	167.7 ± 10.3, *p* < 0.001
BMI (kg/m^2^)		28.1 ± 6.5	28.8 ± 6.2, *p* = 0.001	27.9 ± 5.9, *p* < 0.001
Steatotic liver disease				
	Yes, *n* (%)	1088 (52.0)	645 (52.7)	214 (58.6)
	No, *n* (%)	788 (37.7)	490 (40.0)	151 (41.4)
Mean liver shear stiffness (kPa)		3.5 ± 1.8	3.2 ± 2.0, *p* = 0.04	3.5 ± 1.8, *p* = 0.02
Imaging platform				
	GE HealthCare, *n* (%)	756 (36.2)	262 (21.4)	-
	Philips Healthcare, *n* (%)	113 (05.4)	101 (8.3)	365 (100.0)
	Siemens Healthineers, *n* (%)	1222 (58.4)	861 (70.3)	-

Data are mean ± standard deviation; data in parentheses are percentages

*BMI* body mass index

The training set included 2091 MRI examinations from 1876 patients (with a mean [SD] age of 41.8 [12.5] years and a mean BMI of 28.1 [6.5] kg/m^2^; 984 [52.4%] females). The temporally held-out test set included 1224 MRI examinations from 1135 patients (with a mean [SD] age of 53.9 [13.9] years and a mean BMI of 28.8 [6.2] kg/m^2^; 601 [53.0%] females). The external test set had 365 MRI examinations from 365 patients (with a mean [SD] age of 41.6 [12.6] years and a mean BMI of 27.9 [5.9] kg/m^2^; 185 [50.7%] females).

### Model evaluation using internal multi-site cross-validation

Table 2 shows the performance of the multi-channel model for liver stiffness prediction using different combinations of MRI sequences and EHR data. Using only EHR data, the model achieved an RMSE of 1.59 kPa [95% CI: 1.46, 1.74] and an r of 0.63 [95% CI: 0.58, 0.67]. T2w alone achieved an RMSE of 1.47 kPa [95% CI: 1.35, 1.61] and an r of 0.70 [95% CI: 0.66, 0.73]. Combining T1w, T2w, DWI, and EHR data further improved results to an RMSE of 1.29 kPa [95% CI: 1.19, 1.41] and r of 0.78 [95% CI: 0.75, 0.80].

**Table 2:** Performance of multi-channel model for predicting MRE-based liver shear stiffness using routinely acquired clinical axial T1w, T2w, and DWI MR images, as well as EHR data as inputs

Data	RMSE	MAE	r	${{\boldsymbol{R}}}^{{\boldsymbol{2}}}$
EHR data	1.59 [1.47, 1.74]	1.02 [0.98, 1.07]	0.63 [0.59, 0.67]	0.40 [0.35, 0.45]
DWI	1.64 [1.53, 1.76]	1.05 [1.01, 1.10]	0.60 [0.57, 0.64]	0.37 [0.33, 0.41]
T1w	1.56 [1.45, 1.68]	0.98 [0.94, 1.02]	0.65 [0.61, 0.68]	0.42 [0.38, 0.46]
T2w	1.47 [1.35, 1.61]	0.93 [0.89, 0.97]	0.70 [0.66, 0.73]	0.48 [0.44, 0.53]
T1w + T2w	1.41 [1.31, 1.52]	0.89 [0.85, 0.93]	0.72 [0.70, 0.75]	0.52 [0.48, 0.57]
T1w + T2w + DWI	1.35 [1.25, 1.46]	0.85 [0.82, 0.89]	0.75 [0.72, 0.78]	0.56 [0.52, 0.60]
T1w + T2w + DWI + EHR data	1.29 [1.19, 1.41]	0.81 [0.78, 0.85]	0.78 [0.75, 0.80]	0.60 [0.56, 0.64]

Data in brackets are 95% confidence intervals

*RMSE* root mean squared error (kPa), *MAE* mean absolute error (kPa), *r* Pearson’s correlation coefficient, ${R}^{2}$ coefficient of determination

To evaluate modality contribution, ablation testing compared single- and multi-sequence inputs. As shown in Table 2, combining T1w, T2w, and DWI consistently improved performance relative to any single modality.

### Model evaluation on temporally held-out test set

Tested on the temporally held-out set (Table 3), the model achieved an RMSE of 1.64 kPa [95% CI: 1.40, 1.94] and an r of 0.63 [95% CI: 0.57, 0.70] using EHR data alone. Using T2w data alone yielded better performance than T1w or DWI alone, with an RMSE of 1.53 kPa [95% CI: 1.28, 1.81] and an r of 0.69 [95% CI: 0.65, 0.74]. Combining T1w, T2w, DWI, and EHR data improved the results to an RMSE of 1.37 kPa [95% CI: 1.13, 1.64] and an r of 0.77 [95% CI: 0.73, 0.80].

**Table 3:** Performance of multi-channel models for liver shear stiffness regression using internal test cohort with segmented MRI images and EHR data as inputs

Data	RMSE	MAE	r	${{\boldsymbol{R}}}^{{\boldsymbol{2}}}$
EHR data	1.63 [1.37, 1.94]	0.98 [0.92, 1.06]	0.64 [0.58, 0.70]	0.40 [0.33, 0.49]
DWI	1.67 [1.46, 1.90]	1.06 [1.00, 1.14]	0.63 [0.57, 0.68]	0.40 [0.33, 0.47]
T1w	1.68 [1.38, 2.03]	0.96 [0.89, 1.04]	0.61 [0.57, 0.66]	0.38 [0.33, 0.44]
T2w	1.53 [1.28, 1.81]	0.94 [0.88, 1.01]	0.69 [0.65, 0.74]	0.48 [0.43, 0.54]
T1w + T2w	1.55 [1.34, 1.81]	1.05 [0.99, 1.12]	0.71 [0.67, 0.75]	0.51 [0.45, 0.57]
T1w + T2w + DWI	1.40 [1.19, 1.65]	0.87 [0.81, 0.93]	0.75 [0.71, 0.78]	0.56 [0.51, 0.61]
T1w + T2w + DWI + EHR data	1.37 [1.13, 1.64]	0.82 [0.76, 0.88]	0.77 [0.73, 0.80]	0.59 [0.54, 0.65]

Data in brackets are 95% confidence intervals

*RMSE* root mean squared error (kPa), *MAE* mean absolute error (kPa), *r* Pearson’s correlation coefficient, ${R}^{2}$ coefficient of determination

### Model evaluation on external test set

Tested on the external test set (Table 4), the model achieved an RMSE of 1.37 kPa [95% CI: 1.14, 1.64] and an r of 0.63 [95% CI: 0.55, 0.72] using EHR data alone. Using combined MRI sequences improved the performance to an RMSE of 1.04 kPa [95% CI: 0.91, 1.18] and an r of 0.72 [95% CI: 0.63, 0.79]. The addition of EHR data further improved the performance of the model, achieving an RMSE of 0.99 kPa [95% CI: 0.84, 1.14] and an r of 0.76 [95% CI: 0.69, 0.83].

**Table 4:** Performance of multi-channel models for liver shear stiffness regression using external test cohort with segmented MRI images and EHR data as inputs

Data	RMSE	MAE	r	${{\boldsymbol{R}}}^{{\boldsymbol{2}}}$
EHR data	1.37 [1.14, 1.64]	0.94 [0.85, 1.05]	0.63 [0.55, 0.72]	0.40 [0.30, 0.53]
DWI	1.20 [1.06, 1.36]	0.87 [0.79, 0.96]	0.60 [0.49, 0.69]	0.36 [0.25, 0.48]
T1w	1.20 [1.04, 1.36]	0.79 [0.70, 0.89]	0.61 [0.53, 0.69]	0.37 [0.28, 0.47]
T2w	1.15 [1.00, 1.31]	0.81 [0.73, 0.90]	0.67 [0.59, 0.75]	0.45 [0.34, 0.56]
T1w + T2w	1.09 [0.94, 1.24]	0.75 [0.67, 0.83]	0.68 [0.60, 0.76]	0.47 [0.36, 0.57]
T1w + T2w + DWI	1.04 [0.91, 1.18]	0.74 [0.67, 0.82]	0.72 [0.63, 0.79]	0.52 [0.40, 0.62]
T1w + T2w + DWI + EHR data	0.99 [0.84, 1.14]	0.67 [0.59, 0.74]	0.76 [0.69, 0.83]	0.58 [0.48, 0.68]

Data in brackets are 95% confidence intervals

*RMSE* root mean squared error (kPa), *MAE* mean absolute error (kPa), *r* Pearson’s correlation coefficient, ${R}^{2}$ coefficient of determination

### Impact of liver steatosis

We evaluated the effect of SLD on liver stiffness prediction (Table 5). Multi-channel model performance differed between patients without and with SLD. For patients without SLD, the model achieved an r of 0.76 [95% CI: 0.73, 0.80], while in patients with SLD, performance was slightly lower, with an r of 0.74 [95% CI: 0.70, 0.78].

**Table 5:** Performance of multi-channel model in liver stiffness regression on patients without and with SLD using segmented MRI images and EHR data as inputs

Data	RMSE	MAE	r	${{\boldsymbol{R}}}^{{\boldsymbol{2}}}$
No SLD	1.35 [1.17, 1.57]	0.84 [0.79, 0.90]	0.76 [0.73, 0.80]	0.58 [0.52, 0.64]
SLD	1.36 [1.23, 1.49]	0.85 [0.80, 0.90]	0.74 [0.70, 0.78]	0.55 [0.49, 0.61]

Data in brackets are 95% confidence intervals

*RMSE* root mean squared error (kPa), *MAE* mean absolute error (kPa), *r* Pearson’s correlation coefficient, ${R}^{2}$ coefficient of determination, *SLD* steatotic liver disease

### Error (bias) analysis

**Fig. 3:**
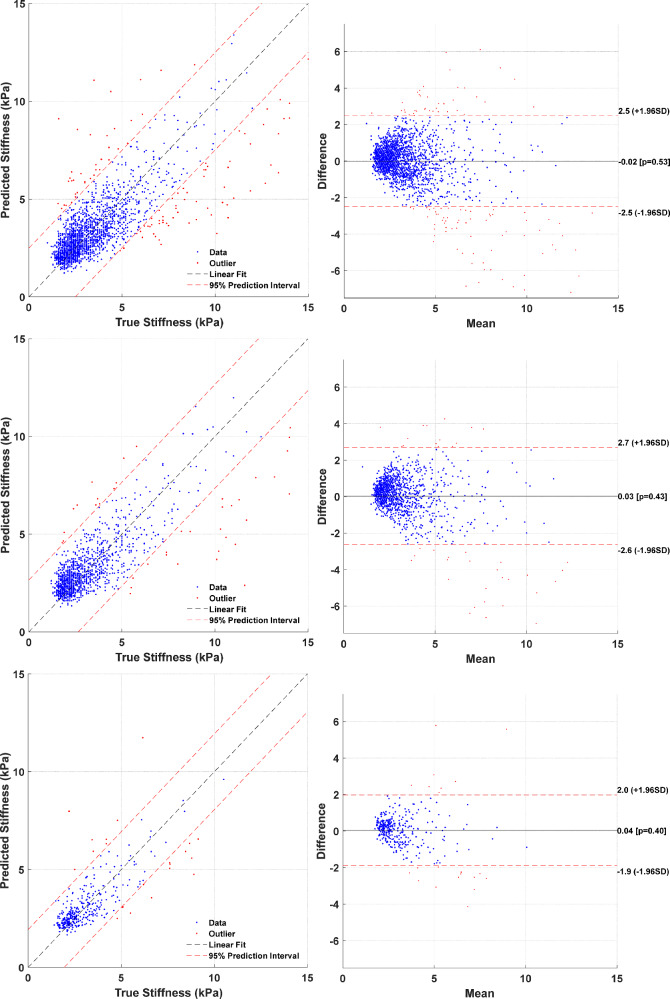
Scatter plots and Bland–Altman analyses for evaluating the performance of the multi-channel model across three datasets. The top row represents the training set, the middle row corresponds to the internal temporally held-out test set, and the bottom row shows the independent held-out external test set

**Fig. 4:**
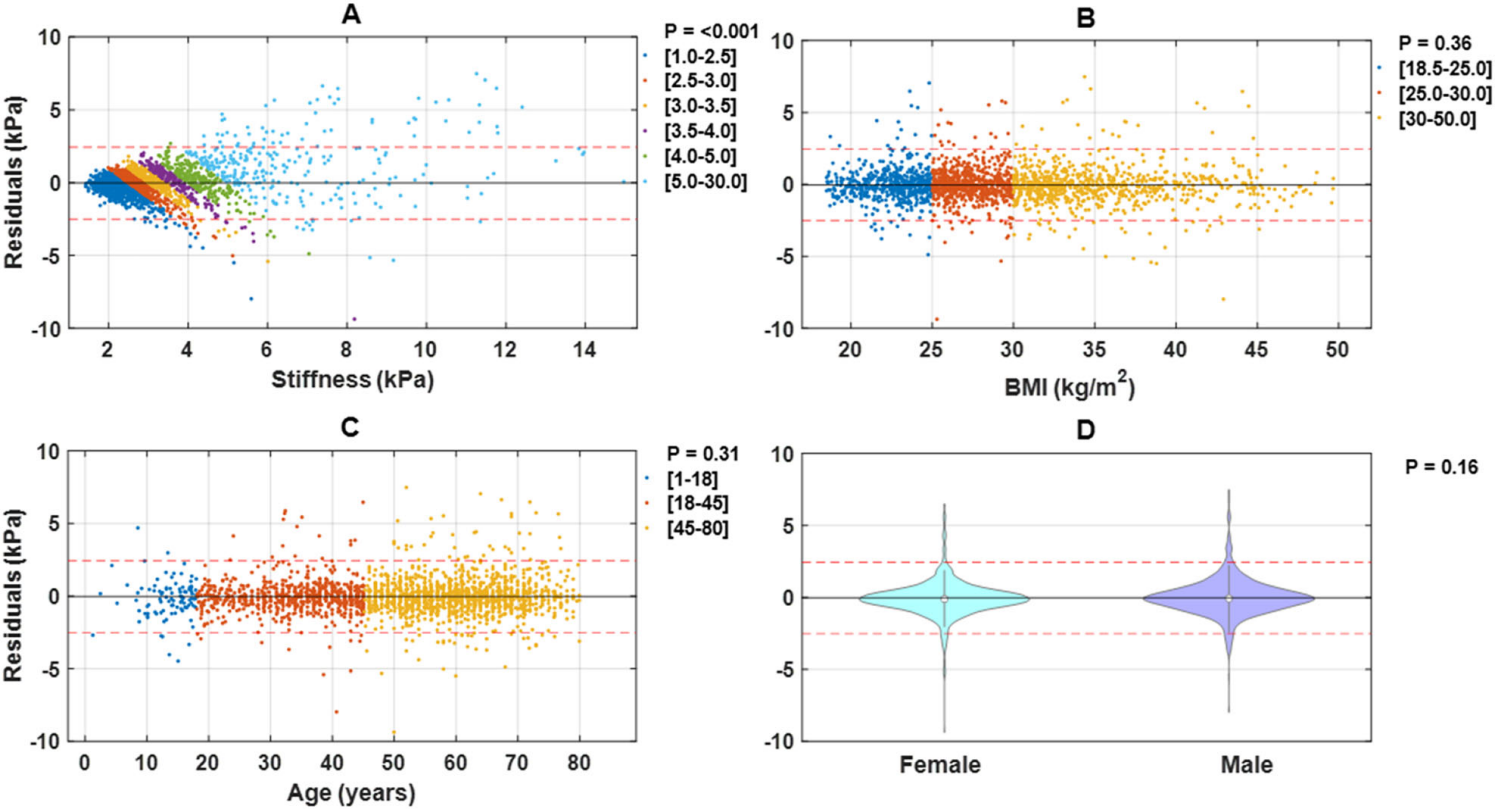
For the training set, residuals are shown for different groups within each category, with *p*-values calculated using ANOVA to assess the level of agreement between residuals across groups. The model demonstrated a bias (*p* < 0.001) for true stiffness, and no significant bias (*p* > 0.05) for BMI, age, and sex. Bland–Altman plots display residuals against **A** MRE-measured liver stiffness, **B** BMI, and **C** age, with *p*-values indicated in the legends. **D** The violin plot shows residuals by sex, where the solid blue line represents the mean residual, and red dashed lines indicate ± 1.96 standard deviations

**Fig. 5:**
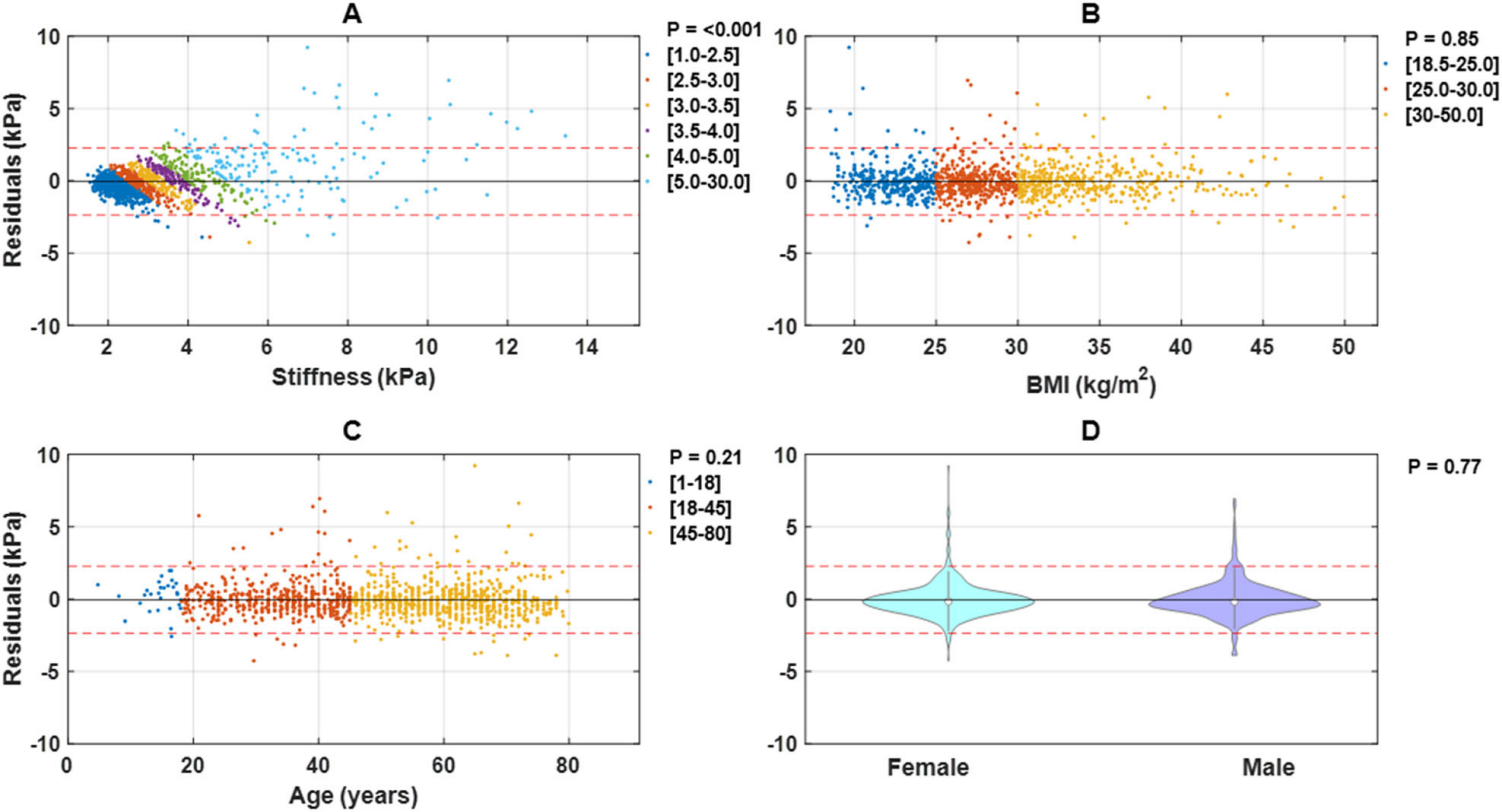
Residuals are shown for different groups within each category for temporally held-out internal test set, with *p*-values calculated using ANOVA to assess the level of agreement between residuals across groups. The model demonstrated a bias (*p* < 0.001) for true stiffness, and no significant bias (*p* > 0.05) for BMI, age and sex. Bland–Altman plots display residuals against **A** MRE-measured liver stiffness, **B** BMI, and **C** age, with *p*-values indicated in the legends. **D** The violin plot shows residuals by sex, where the solid blue line represents the mean residual, and red dashed lines indicate ± 1.96 standard deviations

**Fig. 6:**
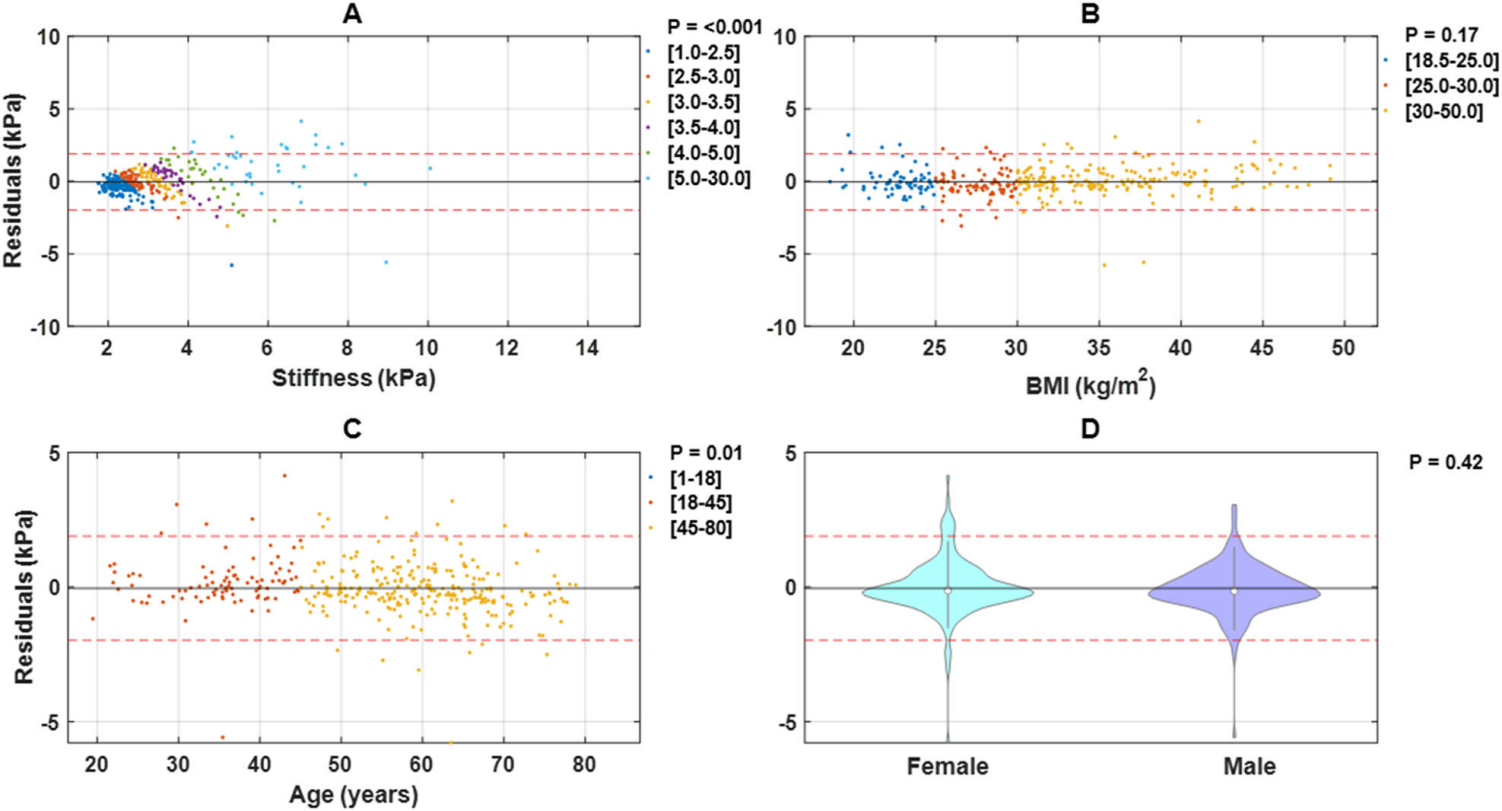
Residuals are shown for different groups within each category for external test set, with *p*-values calculated using ANOVA to assess the level of agreement between residuals across groups. The model demonstrated a bias (*p* < 0.001) for true stiffness, and no significant bias (*p* > 0.05) for BMI, age and sex. Bland–Altman plots display residuals against **A** MRE-measured stiffness, **B** BMI, and **C** age, with *p*-values indicated in the legends. **D** The violin plot shows residuals by sex, where the solid blue line represents the mean residual, and red dashed lines indicate ± 1.96 standard deviations

Figure 3 illustrates the model’s performance across the three datasets using scatter plots and Bland–Altman (BA) analyses. In the training set (first row), the BA plot indicates limits of agreement (LoA) at +2.5 and −2.5 kPa, with a mean bias of 0.02 kPa (*p* = 0.53). In the temporally held-out internal test set (second row), the LoA were +2.7 and −2.6 kPa, with a mean bias of 0.03 kPa (*p* = 0.43). In the external test set (third row), the LoA were narrower at +2.0 and −1.9 kPa, with a mean bias of 0.04 kPa (*p* = 0.40). No systematic bias between predicted and MRE-measured liver stiffness was observed for any dataset (*p* > 0.05). Additional BA plots of residuals stratified by demographic and phenotypical categories for the training, internal, and external datasets are presented in Figs. 4–6. No significant bias was detected by age, sex, or BMI; however, residual magnitude differed across stiffness levels and was greatest for liver stiffness > 6 kPa. This pattern may reflect the relatively small number of advanced fibrosis cases in the training dataset. Expanding training data in this range may improve performance for severe diseases.

## Discussion

This study developed a transformer-based multi-channel deep learning model to predict liver shear stiffness from routine multiparametric MRI and EHR data, potentially reducing the need for dedicated MRE when multiparametric MRI and EHR data are available. This study demonstrates that deep learning models can predict MRE-derived liver stiffness with consistent agreement across internal and external datasets. Importantly, the objective of this work was to predict continuous MRE-derived liver stiffness rather than histology-based fibrosis stage; results should therefore be interpreted in the context of MRE stiffness estimation rather than biopsy-defined staging. In a large, multi-site, multi-vendor combined pediatric and adult cohort, integrating multiple MRI sequences with clinical data yielded correlation coefficients above 0.7 in both internal and external validation. The observed limits of agreement (± 2.5 kPa) are comparable to previously reported inter-scanner and inter-reader variability for liver MRE and US shear-wave elastography studies, which typically fall within ± 2–3 kPa for stiffness values in the range of ≤ ~10–12 kPa [35–38]. While this level of error may limit accurate diagnostic decisions, it is potentially sufficient for initial screening/triage, longitudinal monitoring, and integration with other non-invasive biomarkers to refine fibrosis staging. This approach may offer technical feasibility in settings where dedicated MRE acquisition is limited. Evaluation of cost, time, and workflow impact was beyond the scope of the present study; accordingly, references to accessibility should be interpreted in terms of technical implementation rather than demonstrated economic or operational advantage.

To date, Pollack et al [25] is the only study to predict continuous liver shear stiffness measurements using MRI sequences employed at their institution, including non-contrast T1w LAVA water, LAVA fat, delayed postcontrast T1w LAVA water (120-s delay), and single-shot fast spin-echo T2w images along with EHR data. In their retrospective study of 149 patients, the model achieved an ${R}^{2}$ of 0.50 ± 0.05 using internal 40-fold CV. However, the reliance in part on contrast-enhanced imaging and site-specific MRI protocols in their study limits its applicability across diverse clinical environments, MRI scanners, and patient groups. In comparison, our model demonstrated a better performance across a much larger multi-site, multi-vendor dataset, achieving correlations of 0.78 [95% CI: 0.75, 0.80] in the 10-fold CV (Table 2), 0.77 [95% CI: 0.73, 0.80] in the internal testing (Table 3), and 0.76 [95% CI: 0.69, 0.83] in the external testing (Table 4) experiments. Our model may be preferred as it uses commonly available non-contrast multiparametric MRI sequences that offer several advantages over their contrast-enhanced counterparts, particularly in terms of safety and accessibility. In particular, non-contrast MRI eliminates certain risks related to gadolinium-based contrast agents, such as allergic-like reactions and nephrogenic systemic fibrosis.

A prior study developed a machine learning model using a Support Vector Machine (SVM) classifier with an AUROC of 0.70 for categorically classifying liver shear stiffness (i.e., predicting normal vs. abnormal liver shear stiffness) [15]. This approach, however, used manual liver segmentation and radiomic feature extraction, making it time-intensive and less suitable for routine clinical use. Subsequently, a DL model, DeepLiverNet 1.0 [24], was developed based on a single-institutional cohort that included 273 subjects with known or suspected chronic liver disease. It achieved an AUROC of 0.80 for classifying categorical liver shear stiffness using only T2-weighted images. In that same study, the combination of MRI and EHR data in internal CV led to improved classification performance, with an AUROC of 0.86, compared to AUROCs of 0.83 and 0.80 when using EHR data or imaging data alone. DeepLiverNet 1.0 classified patients with an AUROC of 0.79 in external validation. Our current study presents a multi-channel model, an enhanced transformer-based DL model for predicting continuous liver stiffness measurements from MRI. Developed and validated on a large, multi-site, multi-vendor dataset (*n* = 3591) including T1w, T2w, and DWI sequences, the model achieved stronger correlations with MRE-measured stiffness and offers improved clinical applicability. Across all evaluations, models using combined MRI sequences consistently outperformed those using any single modality, with further gains when EHR data were integrated, reflecting the complementary value of clinical information and imaging features. This multimodal advantage was consistent across datasets, indicating robust performance across patient populations and imaging settings. While formal interpretability analyses were not performed for the present regression framework, prior work using the same cohort to perform liver stiffness classification demonstrated, through Grad-CAM analyses, that model attention was localized to anatomically relevant regions, including the liver and spleen [26]. This prior evidence provides contextual support that the imaging features learned by the model are derived from anatomically relevant regions associated with liver stiffness-related risk stratification.

To gain deeper insight into model behavior, we examined residual patterns. As shown in the Bland–Altman analyses, residual variability remains between predicted and reference stiffness values, which may influence interpretation near diagnostic thresholds. Notably, comparable or greater variability has been reported both within and between systems for ultrasound shear-wave elastography [39]. Residual magnitude increased with stiffness across all validation experiments, though this trend may be of limited clinical relevance above approximately 6–8 kPa, where the liver is already extremely stiff. The model showed no significant effect of age, sex, or BMI on residuals. Although the cohort included both pediatric and adult subjects, the sample size was highly imbalanced (125 pediatric vs. 3554 adult subjects). Given this disparity and differences in age distributions across institutions, pediatric- and adult-specific subgroup analyses were deferred. These will be explored in future work to assess generalizability across age spectra. Model performance showed modest variation in patients with steatotic liver disease and at higher stiffness values (> 6 kPa). This likely reflects altered signal characteristics in the setting of hepatic steatosis, a challenge that has also been reported for MRE itself in prior studies. These findings are consistent with prior reports indicating that liver composition can influence stiffness measurements [32]. This likely reflects the heterogeneous tissue composition in fatty liver, where regions of fat and fibrosis coexist. Such heterogeneity can reduce the accuracy of MRE-derived stiffness values, making model predictions less reliable in this subgroup. Including more SLD cases in training may help improve performance. However, the Fisher z-test showed that the difference in correlations was not statistically significant (z = −1.25, *p* = 0.21), indicating that the model performs comparably between SLD and non-SLD subjects despite the slight numerical difference. These findings highlight the importance of considering patient-specific factors and medical history when developing AI models.

Despite the promising performance of our DL models in predicting liver stiffness, several important limitations should be noted. Owing to the retrospective design, this study does not assess the model’s real-world clinical impact on diagnostic decision-making. Prospective evaluation is the focus of our ongoing work and is beyond the scope of the present work. Histological validation was not feasible given the retrospective, Mult institutional nature of our study, and established fibrosis scoring systems (e.g., METAVIR) were unavailable for most cases. Future efforts will focus on integrating biopsy proven fibrosis grades to enable direct clinico-pathologic correlation and stage-specific validation. The effects of certain potential confounders, such as hepatic iron overload, were not evaluated because relevant data were inconsistently available across participating sites. Additionally, heterogeneity in EHR data quality across institutions may have introduced variability in the completeness and consistency of clinical features used as model inputs. The modeling pipeline relied on automated liver and spleen segmentation to define regions of interest. While segmentation variability constitutes a potential limitation, automated methods substantially reduce interoperator variability compared with manual approaches, improving consistency across cases. The influence of residual segmentation uncertainty on model outputs was not explicitly quantified in this study. Model performance remains moderate, with wider-than-desired 95% limits of agreement (± 2.5 kPa). However, the use of routine MRI and EHR data suggests a favorable cost profile that warrants future health economic evaluation. Further refinements, such as incorporating additional pulse sequences, improving data harmonization, and pursuing prospective validation, may enhance precision and clinical applicability. Finally, while our approach eliminates the need for MRE hardware, we did not perform a formal cost-effectiveness analysis.

In conclusion, our study demonstrates the potential of combining multiparametric abdominal MRI and EHR data to predict continuous liver shear stiffness using advanced DL algorithms, particularly a Swin transformer-based multi-channel model. Using a large, multi-site, and multi-vendor dataset that includes both pediatric and adult patients, we show that integrating diverse MRI sequences with clinical data improves predictive performance. Prospective validation in clinical settings across diverse populations is needed to confirm the model’s utility. Ongoing efforts are also required to reduce prediction errors and further refine the model for greater accuracy.

## Supplementary information

ELECTRONIC SUPPLEMENTARY MATERIAL

## Abbreviations

AI: Artificial intelligence
ANOVA: Analysis of variance
BMI: Body mass index
CI: Confidence interval
CLD: Chronic liver disease
CV: Cross-validation
DL: Deep learning
DWI: Diffusion-weighted imaging
EHR: Electronic health record
FIB-4: Fibrosis-4 score
MASH: Metabolic dysfunction-associated steatohepatitis
MASLD: Metabolic dysfunction-associated liver disease
MRI: Magnetic resonance imaging
NAFLD: Nonalcoholic fatty liver disease
NASH: Nonalcoholic steatohepatitis
PDFF: Proton density fat fraction
RMSE: Root mean squared error
SLD: Steatotic liver disease
T1w: T1-weighted
T2w: T2-weighted
