# Supplementary material for: Development and validation of a deep learning model for liver shear stiffness regression using abdominal multiparametric MRI across multiple sites and vendors
Source: Eur Radiol. 2026 May 13;36(8):6232–45. doi: 10.1007/s00330-026-12448-0 (PMC13341901; doi:10.1007/s00330-026-12448-0)
Supplement: Supplementary file 1 — ELECTRONIC SUPPLEMENTARY MATERIAL [file 330_2026_12448_MOESM1_ESM.pdf]

Development and validation of a deep learning model for liver shear stiffness regression using abdominal multiparametric MRI across multiple sites and vendors

ELECTRONIC SUPPLEMENTARY MATERIAL

S.1.1 MRI Acquisition Parameters

MRI examinations were acquired using routine clinical abdominal MRI protocols at all participating sites (Cincinnati Children's Hospital Medical Center (CCHMC), New York University (NYU), the University of Wisconsin (UW), and University of Michigan (UM)). Sequences included non-contrast 3D T1-weighted gradient echo fat-suppressed sequences such as LAVA, VIBE, THRIVE, or Dixon water reconstructions; axial T2-weighted fast spin-echo fat-suppressed sequences; and axial diffusion-weighted imaging (DWI) spin-echo echo-planar sequences at multiple b-values. Parameter ranges, including field of view (FOV), slice thickness (ST), echo time (TE), and repetition time (TR) for each sequence, stratified by vendor and field strength, are summarized in **Table S1**. **Table S2** presents a summary of the MRI systems utilized in our study, organized by manufacturers and models. Following this general overview of MRI acquisition parameters across all participating sites, detailed site-specific acquisition characteristics are provided below.

CCHMC — All MRI examinations were performed using MRI systems from one of two manufacturers, GE HealthCare 1.5T (n=83 examinations) and 3.0T (n=7 examinations) and Philips Healthcare 1.5T (n=181 examinations) and 3.0T (n=33 examinations). Abdominal MRI protocols included the following sequences: axial T1w gradient echo fat-saturated (echo time ms/ repetition time ms, 1.5T [3.0 (1.7 - 3.3)/ 6.3 (3.7 - 7.3)], 3.0T [1.8 (1.7 - 2.0)/ 6.0 (4.7 - 7.5)]; slice thickness, 1.5T [4.2 (4.0 - 5.0) mm], 3.0T [4.3 (4.0 -

5.9) mm]; field of view, 1.5T [34.3 (25.0 - 44.0) cm], 3.0T [34.9 (30.0 - 42.0) cm]) and axial T2w fast spin-echo fat-saturated (echo time ms/ repetition time ms, 1.5T [71.0 (53.0 - 96.0)/ 9819.0 (2857.1 - 27272.7)], 3.0T [84.4 (69.2 - 110.2)/ 11559.7(4000.0 - 16666.7)]; slice thickness, 1.5T [5.0 (4.0 - 6.5) mm], 3.0T [5.1 (5.0 - 6.0) mm]; field of view, 1.5T [33.7 (23.0 - 44.0) cm], 3.0T [34.6 (30.0 - 40.0) cm]) sequences. For additional details about DWI (b-values 500-800) and Philips Healthcare MRI acquisition parameters see **Table S1.**

NYU. — All MRI examinations were performed using MRI systems from a single manufacturer, Siemens Healthcare 1.5T (n=1,587 examinations) and 3.0T (n=496 examinations). Abdominal MRI protocols included the following sequences: axial T1w gradient echo fat-saturated (echo time ms/ repetition time ms, 1.5T [1.9 (1.3 - 2.4)/ 4.9 (2.6 - 6.8)], 3.0T [1.3 (1.0 - 1.6)/ 3.8 (2.6 - 4.1)]; slice thickness, 1.5T [3.0 (2.5 - 3.2) mm], 3.0T [3.0 (2.7 - 3.5) mm]; field of view, 1.5T [36.3 (27.5 - 45.0) cm], 3.0T [37.6 (30.0 - 45.0) cm]) and axial T2w fast spin-echo fat-saturated (echo time ms/ repetition time ms, 1.5T [96.3 (82.0 - 108.0)/ 2843.3 (1390.0 - 11260.0)]; 3.0T [101.2 (74.0- 105.0)/ 3399.2 (2120.0 - 8495.7)]; slice thickness, 1.5T [4.4 (4.0 - 6.0) mm], 3.0T [4.0 (4.0 - 5.0) mm]; field of view, 1.5T [34.6 (25.0 - 45.0) cm], 3.0T [36.4 (30.0 - 44.0) cm]) sequences. Refer to **Table S1** for more information on the DWI (b-value 800) and Siemens Healthineers MRI acquisition parameters.

UW. — All MRI examinations were performed using MRI scanners from one manufacturer, GE HealthCare 1.5T (n=338 examinations) and 3.0T (n=590 examinations). Abdominal MRI protocols included the following sequences: axial T1w gradient echo fat-saturated (echo time ms/ repetition time ms, 1.5T [1.8 (1.6 - 5.8)/ 3.8

(3.4 - 14.9)], 3.0T [1.9 (1.4 - 2.4)/ 4.0 (3.2 - 5.2))]; slice thickness, 1.5T [4.6 (3.9 - 8.0) mm], 3.0T [3.6 (3.2 - 8.0) mm]; field of view, 1.5T [38.0 (30.0 - 50.0) cm], 3.0T [37.9 (24.0 - 48.0) cm]) and axial T2w fast spin-echo fat-saturated MRI (echo time ms/ repetition time ms, 1.5T [92.3 (88.1 - 98.0)/ 11433.0 (2000.0 - 24000.0)], 3.0T [88.9 (23.4 - 110.3)/ 11930.0 (2200.0 - 25714.3)]; slice thickness, 1.5T [5.0 (5.0 - 6.0) mm], 3.0T [4.9 (3.0 - 6.0) mm]; field of view, 1.5T [37.1 (32.0 - 48.0) cm], 3.0T [36.9 (24.0 - 50.0) cm]) sequences. For further information on the DWI (b-value 500) and GE HealthCare MRI acquisition parameters, please see **Table S1**.

UM. — All MRI examinations were performed using MRI systems from one manufacturer, Philips Healthcare 1.5T (n=360 examinations) and 3.0-T (n=5 examinations). Abdominal MRI protocols included the following sequences: axial T1w gradient echo fat-saturated (echo time ms/ repetition time ms, 1.5T [1.8 (1.5 - 1.9)/ 3.9 (3.5 - 4.0)], 3.0T [1.4 (1.4 - 1.4)/ 3.0 (3.0 - 3.0)]; slice thickness, 1.5T [4.5 (4.0 - 5.5) mm], 3.0T [4.0 (4.0 - 4.0) mm]; field of view, 1.5T [36.1 (27.0 - 46.0) cm], 3.0T [38.6 (33.0 - 40.0) cm]) and axial T2w fast spin-echo fat-saturated (echo time ms/ repetition time ms, 1.5T [80.8 (80.0 - 90.0)/ 2229.7 (1449.1 - 6928.5)], 3.0T [77.7 (77.6 - 78.0)/ 2725.0 (2725.0 - 2725.0)]; slice thickness, 1.5T [6.0 (5.0 - 7.0) mm], 3.0T [6.0 (6.0 - 6.0) mm]; field of view, 1.5T [36.3 (26.0 - 46.5) cm], 3.0T [39.0 (35.0 - 42.0) cm]) sequences. See **Table S1** for detailed information on the DWI (b-values 600 and 800) and Philips Healthcare MRI acquisition parameters.

S.1.2. MRE Acquisition Parameters

Liver shear stiffness was quantified using MRE performed on 1.5T or 3T MRI scanners across the study sites. External low-frequency mechanical vibrations (60 Hz)

were transmitted to the liver via an active-passive acoustic driver system, inducing shear waves in the hepatic tissue. A modified phase-contrast gradient-echo MRI sequence was used to capture shear wave-related tissue displacement, and the resulting data were processed using an inversion algorithm to generate elastograms. Liver stiffness values were then computed locally at each site and reported in clinical imaging reports in kilopascals (kPa).

S.1.3. EHR Data

A variety of demographic and clinical variables were collected from the electronic health records (EHR) at each study site, as shown in **Table S3**. Notably, the EHR input included derived laboratory indices such as APRI and FIB-4 score, allowing the model to implicitly incorporate established serum fibrosis biomarkers into its prediction framework.

Supplemental Methods

S.2.1. Segmentation of liver and spleen

We used the MRSegmentator segmentation model (29) to automatically segment the liver and spleen from axial T1w, T2w, and DWI MR images. For DWI, we extracted the volume of the lowest b-value for organ segmentation. A low b-value means minimal diffusion sensitization, so the signal is primarily determined by T2 relaxation properties, and water diffusion has little impact on image contrast. Consequently, these images appear similar to T2w images. Thus, MRSegmentator is able to perform segmentation successfully on the DWI volume with low b-values. Afterward, we matched the liver and spleen masks from the lowest b-value to imaging volumes of other b-values.

S.2.2. Multi-channel deep learning models

Our multi-channel model includes separate input channels for imaging and EHR data. The imaging inputs are 3D segmented volumes of liver and spleen from axial T1w, T2w, and DWI MR images, each with a size of 224 × 224 × the number of slices. For feature extraction, we utilized a publicly available, pretrained Swin Transformer model (trained on ImageNet), which was used without fine-tuning. Because the Swin Transformer expects 3-channel RGB input, each grayscale MRI slice was replicated across three channels to form pseudo-RGB images. The Swin Transformer served as a fixed feature extractor to generate latent representations from the segmented liver and spleen images for each sequence (T1w, T2w, and DWI). These features were aggregated using average pooling across slices to generate a single embedding vector per modality. The input of the EHR channel comprised a structured vector of *k* EHR data. Deep features extracted from each imaging sequence were processed through trainable dense layers, while EHR features passed through a separate dense layer. The resulting representations were fused and passed to a final linear output layer to predict continuous liver shear stiffness values.

We conducted a comprehensive hyperparameter optimization for our regression model using a 10-fold CV, focusing on parameters such as learning rate, batch size, and network depth using grid search. This optimization process was integrated into the training phase to enhance model accuracy and convergence. The final configuration for training the multi-channel model (**Figure S1**) included 100 epochs with a base learning rate of 0.0001, utilizing the Adam optimizer (learning rate=0.0001, learning rate decay factor=0.1 every 10 epochs). We employed regularization techniques such as an early

stopping mechanism, to mitigate overfitting and enhance generalization. The early stopping criterion was based on monitoring the validation loss, and training was terminated if no improvement was observed over five consecutive epochs. All experiments were executed on a high-performance workstation equipped with 256 GB of RAM and dual NVIDIA RTX A4500 GPUs.

Table S1 MRI Acquisition Parameters.

Site	Sequence	Vendor	*B*	FOV (cm)	ST (mm)	TE (ms)	TR (ms)
CCHMC	T1w	GE	1.5T	34.3 (25.0 - 44.0)	4.2 (4.0 - 5.0)	3.0 (1.7 - 3.3)	6.3 (3.7 - 7.3)
			3.0T	34.9 (30.0 - 42.0)	4.3 (4.0 - 5.9)	1.8 (1.7 - 2.0)	6.0 (4.7 - 7.5)
		Philips	1.5T	33.6 (22.0 - 45.0)	3.9 (3.0 - 5.0)	N/A	5.6 (4.0 - 8.8)
			3.0T	34.1 (26.0 - 45.0)	3.7 (3.0 - 4.0)	N/A	4.1 (3.6 - 5.3)
	T2w	GE	1.5T	33.7 (23.0 - 44.0)	5.0 (4.0 - 6.5)	71.0 (53.0 - 96.0)	9819.0 (2857.1 - 27272.7)
			3.0T	34.6 (30.0 - 40.0)	5.1 (5.0 - 6.0)	84.4 (69.2 - 110.2)	11559.7 (4000.0 - 16666.7)
		Philips	1.5T	33.6 (22.0 - 45.0)	5.0 (4.0 - 6.0)	80.1 (53.0 - 120.0)	2450.5 (319.4 - 8571.4)
			3.0T	34.1 (26.0 - 45.0)	4.9 (4.0 - 6.0)	79.4 (74.9 - 83.4)	2725.0 (2725.0 - 2725.0)
	DWI (b-values 500/800)	GE	1.5T	35.4 (24.0 - 46.0)	5.8 (5.0 - 8.0)	69.9 (64.0 - 86.1)	9170.6 (3000.0 - 40000.0)
			3.0T	34.3 (30.0 - 40.0)	6.7 (5.0 - 8.0)	68.1 (61.3 - 71.7)	6552.1 (2500.0 - 15000.0)
		Philips	1.5T	33.8 (22.0 - 45.0)	5.7 (5.0 - 6.0)	62.4 (60.1 - 77.0)	1385.7 (832.6 - 6666.7)
			3.0T	34.0 (26.0 - 45.0)	4.9 (4.0 - 5.0)	69.5 (63.5 - 79.3)	2884.6 (1274.5 - 4900.0)
NYU	T1w	Siemens	1.5T	36.3 (27.5 - 45.0)	3.0 (2.5 - 3.2)	1.9 (1.3 - 2.4)	4.9 (2.6 - 6.8)
			3.0T	37.6 (30.0 - 45.0)	3.0 (2.7 - 3.5)	1.3 (1.0 - 1.6)	3.8 (2.6 - 4.1)
	T2w		1.5T	34.6 (25.0 - 45.0)	4.4 (4.0 - 6.0)	96.3 (82.0 - 108.0)	2843.3 (1390.0 - 11260.0)
			3.0T	36.4 (30.0 - 44.0)	4.0 (4.0 - 5.0)	101.2 (74.0-105.0)	3399.2 (2120.0 - 8495.7)
	DWI (b-value 800)		1.5T	34.8 (25.8 - 45.0)	8.0 (4.0 - 8.0)	65.3 (52.0 - 106.0)	4006.2 (1900.0 - 12500.0)
			3.0T	37.4 (30.0 - 44.0)	5.5 (5.0 - 6.5)	65.8 (59.0 - 90.0)	6413.1 (2900.0 - 10200.0)
UW	T1w	GE	1.5T	37.9 (30.0 - 50.0)	4.6 (3.9 - 8.0)	1.8 (1.6 - 5.8)	3.8 (3.4 - 14.9)
			3.0T	37.8 (30.0 - 48.0)	3.6 (3.2 - 8.0)	1.9 (1.4 - 2.2)	4.1 (3.2 - 4.8)
	T2w		1.5T	37.0 (32.0 - 46.0)	5.0 (5.0 - 5.0)	92.2 (88.1 - 98.0)	11413.0 (2000.0 - 20000.0)
			3.0T	36.7 (30.0 - 50.0)	4.9 (3.0 - 5.0)	88.8 (80.9 - 107.0)	11985.1 (2200.0 - 25714.3)
	DWI (b-value 500)		1.5T	38.2 (32.0 - 46.0)	5.0 (4.0 - 6.0)	60.6 (58.7 - 70.9)	15459.2 (2500.0 - 36000.0)
			3.0T	37.7 (32.0 - 50.0)	5.0 (4.0 - 6.0)	54.2 (45.6 - 74.0)	12121.0 (3157.9 - 30000.0)
UM	T1w	Philips	1.5T	36.1 (27.0 - 46.0)	4.5 (4.0 - 5.5)	1.8 (1.5 - 1.9)	3.9 (3.5 - 4.0)
			3.0T	38.6 (33.0 - 40.0)	4.0 (4.0 - 4.0)	1.4 (1.4 - 1.4)	3.0 (3.0 - 3.0)
	T2w		1.5T	36.3 (26.0 - 46.0)	6.0 (5.0 - 7.0)	80.8 (80.0 - 88.0)	2229.7 (1449.1 - 6928.5)
			3.0T	39.0 (35.0 - 42.0)	6.0 (6.0 - 6.0)	77.7 (77.6 - 78.0)	2725.0 (2725.0 - 2725.0)

	DWI (b-values 600/800)		1.5T	36.7 (26.0 - 47.5)	6.0 (5.0 - 7.0)	63.5 (56.4 - 79.5)	1763.8 (1093.7 - 2984.2)
			3.0T	37.6 (33.0 - 40.0)	6.0 (6.0 - 6.0)	69.1 (68.8 - 70.0)	1585.2 (1493.8 - 1707.2)

Note. —Data are means, with ranges in parentheses. *B* = magnetic field strength, FOV = field of view, ST = slice thickness, TE = echo time, TR = repetition time.

Table S2 shows the different MRI systems used in the study, categorized by manufacturers.

**Manufacturer**	**Model**
Philips Healthcare	Ingenia (1.5T)<br>Ingenia (3.0T)<br>Ingenia Ambition X (1.5T)<br>Ingenia Elition X (3.0T)
GE HealthCare	SIGNA Architect (3.0T)<br>Optima MR450w (1.5T)<br>Signa HDxt (1.5T)<br>DISCOVERY MR750 (3.0T)<br>DISCOVERY MR750w (3.0T)<br>SIGNA Artist (1.5T)<br>SIGNA Premier (3.0T)<br>SIGNA HDx (1.5T)<br>SIGNA PET/MR (3.0T)
Siemens Healthineers	Aera (1.5T)<br>Skyra (3.0T)<br>MAGNETOM Vida (3.0T)<br>MAGNETOM Sola (1.5T)<br>Prisma_fit (3.0T)<br>Biograph_mMR (3.0T)<br>Symphony (1.5T)

Table S3 Summary of electronic health record (EHR) data categories and variables collected across multiple sites, including biometrics, demographics, medical history, laboratory results, and MRI-related data. Missing data are noted.

Category	Included	Excluded
Biometrics (n = 1)	BMI	Height, weight, blood pressure (diastolic/systolic)
Demographics (n = 4)	Gender, age, race, ethnicity	DOB
History (diagnoses, n = 4)	Alcohol Liver Disease, Diabetes Type2, Hepatitis B/C, Steatotic Liver Disease	Autoimmune Hepatitis(AIH), Fontan, Hepatic Fibrosis, Liver Transplantation, Primary Biliary Cirrhosis(PBC), Primary Sclerosing Cholangitis(PSC)
Lab (n = 9)	Albumin (g/dL), Alkaline phosphatase (U/L), ALT (U/L), AST (U/L), Direct bilirubin (mg/dL), Total bilirubin (mg/dL), Glucose (mg/dL), Hematocrit (%), Platelet count (K/uL)	GGT(U/L), A1C(%)
MRI related (n = 1)	Liver fat fraction (PDFF) ≥6%	Liver volume, liver stiffness, R2 & T2 (iron-related)

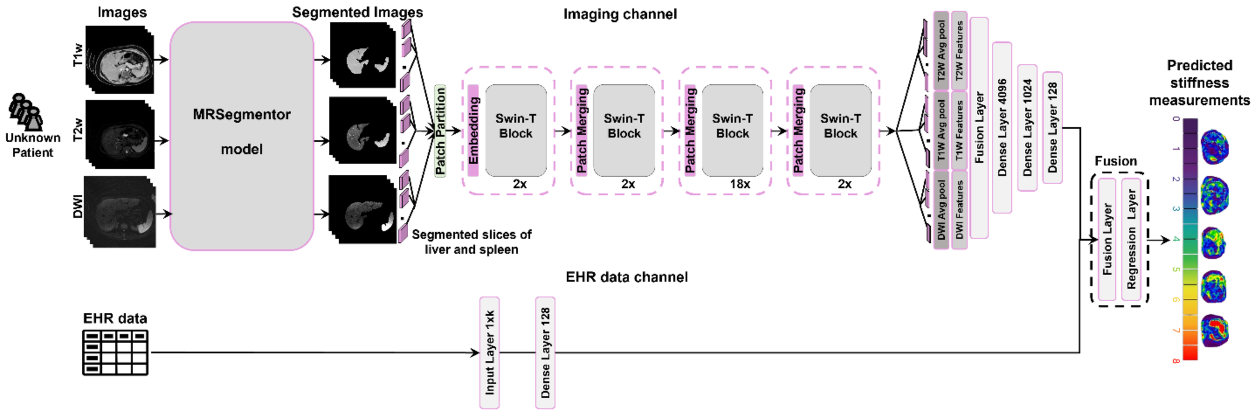

Figure S1: An overview of the architecture of our multi-channel transformer-based DL model which is designed to effectively process and segment MR images, starting with the input imaging layer that integrates three distinct input channels including T1w, T2w, and DWI images. The MRSegmentator was used to segment the liver and spleen from input MR images, which were resized to a standardized 224 x 224 resolution. To extract and analyze the unique latent features within these segmented liver and spleen images, we incorporated a pre-trained Swin Transformer, which captures the latent image features. An adaptive learning block featuring trainable layers was used to enhance the model's capacity to learn and leverage individual latent features.
